# Dietary Fiber and Melanoma: Exploring Microbiome-Driven Immune Modulation

**DOI:** 10.3390/cancers18020203

**Published:** 2026-01-08

**Authors:** Laci Turner, Connor K. Sisk, Nabiha Yusuf

**Affiliations:** 1 Heersink School of Medicine, University of Alabama at Birmingham, 1670 University Blvd, Birmingham, AL 35233, USA; lacimt@uab.edu (L.T.); sisk@uab.edu (C.K.S.); 2 Department of Dermatology, University of Alabama at Birmingham, 1670 University Blvd., VH566A, Birmingham, AL 35294, USA

**Keywords:** melanoma, dietary fiber, prebiotics, gut microbiome, short-chain fatty acids, immune modulation, immune checkpoint inhibitors, beta-glucan, inulin, microbiome modulation

## Abstract

**Background/Objectives**: The gut microbiome influences melanoma biology and response to immune checkpoint inhibitors. Dietary fiber is a key modifiable factor that shapes the microbial composition and metabolite production. This review summarizes mechanistic, preclinical, and clinical evidence describing how fiber and fiber-responsive taxa may affect melanoma immunity and treatment outcomes. **Methods**: A literature search of MEDLINE, Embase, and Scopus identified studies published within the past five years examining dietary fiber, gut microbiome interactions, immune modulation, or melanoma outcomes. After screening 491 unique records, 49 peer-reviewed mechanistic, preclinical, observational, and interventional studies were synthesized qualitatively in this narrative review. **Results**: Fiber fermentation produces short-chain fatty acids that regulate dendritic cell activation, T-cell priming, and cytokine signaling. Preclinical melanoma models show that fibers such as inulin and β-glucan enhance IFN-γ-driven antitumor immunity, increase CD8^+^ infiltration, and improve checkpoint blockade efficacy in a microbiota-dependent manner. In humans, fiber-rich diets and enrichment of taxa such as *Bifidobacterium*, *Faecalibacterium*, and *Akkermansia* are associated with improved PD-1 inhibitor responses, longer progression-free survival, and possible reductions in ICI-related colitis. Although epidemiologic studies suggest no clear association between fiber intake and melanoma incidence, dietary fiber intake appears to correlate strongly with treatment-related outcomes. **Conclusions**: Dietary fiber represents a potentially safe and plausible adjunct to melanoma immunotherapy. However, study variability and emerging counterevidence highlight the need for controlled trials to clarify causality and define optimal fiber-based interventions.

## Simple Summary

Melanoma pathophysiology is strongly influenced by the immune system, and a growing body of evidence suggests that the gut microbiome plays a significant role in how patients respond to treatment. Dietary fiber can be fermented into metabolites that impact immune function, strengthen the gut barrier, and promote the growth of bacterial species linked to better responses to immunotherapy. This review summarizes the current literature surrounding the impact of dietary fiber on the gut microbiome and melanoma while highlighting preclinical and clinical studies investigating the role of dietary fiber interventions in melanoma pathogenesis, therapeutic responsiveness, and outcomes.

## 1. Introduction

The gut microbiome (GM) plays an important role in the development of the immune system and responsiveness to various pathogens and disease processes. Consisting of bacteria, fungi, viruses, and protozoans, an estimated 70% of host immune cells dwell within and in close proximity to the gut [1]. This relationship is characterized by constant streams of communication between the diverse microbial populations and the host immune system. This dynamic system enables a complex interplay that maintains a role in the regulation of systemic inflammation and immune responses.

Importantly, this dynamic is not unidirectional; changes in the immune system strongly influence gut microbial composition. For example, systemic infection from atypical or non-commensal organisms or inflammation may disrupt the GM and lead to dysbiosis, a disadvantageous imbalance in the normal interplay between host immunity and the GM [2]. Dysbiosis may be caused by antibiotics, normal aging, smoking, hormonal changes, other offending agents, and even many anticancer therapies, which can alter GM composition through off-target cytotoxic mechanisms and disrupt gut barrier integrity [3,4]. Further evidence of the bidirectionality of this relationship manifests in the interactions between the GM and various cancer therapeutics; the GM not only is impacted directly by cancer therapeutics but also influences the effectiveness and toxicity of these treatments both within and outside of the gastrointestinal (GI) tract [5,6]. Overall, disruptions to this complex balance can drive changes within the immune system, leading to systemic inflammation, cancer tumorigenesis, and disease progression [3].

Given the importance of effective host antitumor responses for melanoma outcomes, impairment of a healthy GM–immune system axis can have significant implications for melanoma progression and treatment outcomes [7]. Alterations in the gut microbial composition can precipitate changes in antigen presentation, cytokine signaling, and T-cell activation. For instance, gut microbes influence antitumor immunity by engaging pattern recognition receptors (PRRs), initiating downstream pathways that enhance immune activation [8]. Microbial stimulation of the stimulator of interferon (IFN) genes (STING) pathway, a type of PRR, has been shown to induce type I IFNs in dendritic cells (DC) [9]. Activation of these receptors increases cytokine signaling, promotes tumor cell death, and improves antigen-presenting cell recruitment, collectively strengthening T-cell priming and overall antitumor responses [10]. The GM and its metabolites can induce signals that affect DCs, natural killer (NK) cells, B cells, CD8^+^ T cells, regulatory T (Treg) cells, and other immune cells [11]. Through these mechanisms, the GM shapes interactions between the tumor microenvironment (TME) and the immune system, influencing responsiveness to melanoma therapies such as immune checkpoint inhibitors (ICIs) [12,13].

On this basis, the GM and the mechanisms behind its disruption have emerged as a new frontier in melanoma research, particularly their role in influencing disease incidence, progression, therapeutic responsiveness, and immune-related adverse events (irAEs). For instance, certain microbial species have been associated with different stages in melanoma progression. Compared to patients with late-stage melanoma, those with early-stage melanoma are more likely to have greater microbial diversity and a higher abundance of certain bacteria, such as *Roseburia* [14]. Another study by Gopalakrishnan et al. found that responders to anti-PD-1 therapy had significantly higher alpha diversity, the diversity and spatial distribution of a given microbial sampling, compared to non-responders (*p* < 0.01), and patients with the highest alpha diversity experienced the longest progression-free survival (PFS) [15,16]. However, these findings have not been consistent across populations, as other cohorts have reported decreased alpha diversity in responders compared to non-responders (*p* < 0.05), or found no significant differences in alpha diversity between response groups [17,18,19]. Together, these findings suggest that microbial signatures may differ across populations and stages of melanoma progression. A more thorough investigation of the underlying mechanisms may be necessary to characterize this complex dynamic.

Diet is one of the most significant modifiers of the GM, and within diet, many nutrients influence microbial diversity and mechanisms of immune function. Among these, dietary fiber has received increasing amounts of attention due to its role in microbial diversity, immune response, and intestinal homeostasis [20]. Examining the impact of dietary fiber and the mechanisms by which fiber-centered dietary changes modulate the GM may offer insight into potentially modifiable factors that shape immunotherapy response.

The purpose of this narrative review is to synthesize current mechanistic, preclinical, and clinical evidence describing how dietary fiber and fiber-driven changes in the GM may influence melanoma biology. Specifically, it examines proposed mechanistic pathways, evaluates findings from relevant preclinical and clinical trials, summarizes observational and interventional data in humans, and highlights how dietary fiber may shape responses to ICIs and immunotherapy-related toxicity. Together, these components aim to provide an integrated understanding of the potential role of dietary fiber as an adjunct to melanoma treatment.

## 2. Materials and Methods

A comprehensive literature search was conducted using MEDLINE (via PubMed), Embase, and Scopus to identify studies examining the relationship between dietary fiber, GM composition, immune modulation, and clinical outcomes in melanoma. The literature search included relevant peer reviewed articles published between 2003 and 2025 and was limited to studies published in English. Foundational studies were included to help provide biological context, along with clinical trials, observational studies, and translational investigations focused on dietary fiber, gut microbiome modulation and immunotherapy outcomes. Conference abstracts, non-peer-reviewed materials, and abstract-only publications were excluded. Complete search strategies with MeSH terms, keywords, Boolean operators, and other filters for Embase, MEDLINE, and Scopus can be provided upon request.

**Figure 1:**
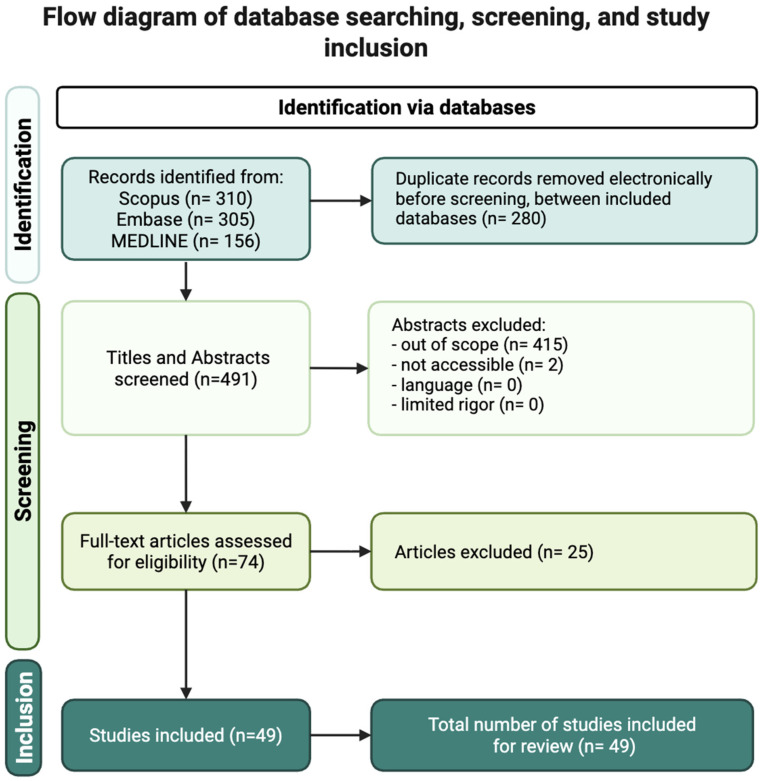
Flowchart showing the literature search and study selection process, including databases surveyed, title and abstract screening, full-text review, and final inclusion.

Across databases, the following totals were retrieved: 156 records from MEDLINE, 305 from Embase, and 310 from Scopus, resulting in 771 total references. After removal of 280 duplicate records, 491 unique articles remained and were imported into Covidence (Veritas Health Innovation, Melbourne, Australia), a systematic review management platform (Figure 1).

Title and abstract screening were completed independently by two reviewers with in-person collaborative meetings for resolving disagreements when necessary. All 491 articles underwent title/abstract screening. After this stage, 74 studies met eligibility criteria for full-text evaluation. Full-text review was conducted by two additional reviewers. Following application of inclusion and exclusion criteria, 49 studies were identified as eligible for qualitative synthesis and data extraction for this narrative review (Figure 1).

Inclusion criteria included articles published within the past five years relevant to dietary fiber, the GM, immune modulation, or melanoma outcomes; peer-reviewed clinical trials and original research studies; and articles available in English with full-text accessibility. Exclusion criteria included articles without full-text availability, conference abstracts, posters or presentations, non-peer-reviewed sources, and publications not directly related to dietary fiber, microbiome interactions, or melanoma (Figure 1). Because this review aims to synthesize both mechanistic and clinical findings, studies were included even if fiber was one component of a broader dietary or microbiome-modulating intervention, as long as the outcomes were relevant to melanoma or immune function.

## 3. Discussion

### 3.1. Dietary Fiber Overview

Dietary fiber is broadly defined as the indigestible carbohydrate components of plants and is categorically classified as a prebiotic, which are indigestible substrates that are broken down by gut bacteria and used for energy, primarily in the large intestine [21]. Formally, prebiotics were defined as substrates that are selectively utilized by host microorganisms to confer a health benefit [22]. While there are many types of prebiotics, dietary fiber represents the most common form and is selectively utilized by host microorganisms to improve host health [23]. Dietary fiber is found in many plant-derived foods, such as vegetables, fruits, oats, barley, and seeds, in a variety of subtypes. For example, fruits contain high amounts of pectin, while whole grains contain large amounts of cellulose and hemicellulose [24]. Additionally, most prebiotics, including dietary fibers, are readily and safely available to patients and general consumers alike as over-the-counter supplements.

Dietary fiber is generally categorized into two forms: soluble and insoluble. Soluble fibers undergo fermentation by colonic bacteria to produce short-chain fatty acids (SCFAs) like butyric, acetic, and propionic acids, which in turn provide energy to both the human host and microbiota [25]. Common examples of SCFAs include pectins, inulin (a fructooligosaccharide), xyloglucan, fructooligosaccharides, and galactooligosaccharides [24]. Insoluble fibers, such as cellulose, lignin, and resistant starches, cannot be fermented and help bulk stool. These metabolites enter the systemic circulation and behave as ligands for specific G-protein-coupled receptors (GPCRs), mechanisms which are directly implicated in certain immune system functions and oncogenic disease processes (Table 1) [25].

SCFAs, particularly butyrate, act through receptor-mediated and epigenetic effects. While healthy colonocytes preferentially use butyrate as an energy source, cancer cells rely more heavily on glycolysis, causing unmetabolized butyrate to accumulate in the nucleus. This buildup inhibits histone deacetylase (HDAC) activity and increases histone acetylation, promoting tumor-suppressor gene expression through enhanced histone acetyltransferase (HAT) function [31]. Although these mechanisms have primarily been characterized in colorectal and lymphoma models, they describe a rationale for how fiber-derived metabolites may influence commonalities in tumor biology that might also apply to melanoma.

Dietary fiber regulates gut microbial substrate use, mucosal barrier stability, and downstream immune pathways relevant to tumor control. Under fiber deprivation, gut bacteria increasingly catabolize host glycoproteins, resulting in measurable erosion of the colonic mucosal barrier in mouse models [32]. Distinct fibers enrich specific microbial taxa, such as the inulin-driven expansion of *Bifidobacterium* and *Faecalibacterium* species, which have been associated with enhanced antitumor immunity and responsiveness to ICIs [33]. Epidemiologic links between fiber intake and reduced colorectal carcinoma risk further highlight the systemic oncologic implications of fiber-microbiome interactions [34,35].

### 3.2. Immune and Microbiome-Mediated Mechanisms Linking Fiber, the Gut Microbiome, and Melanoma

#### 3.2.1. Mechanisms of Immunomodulation by Dietary Fiber

Dietary fiber influences immune pathways relevant to melanoma primarily through effects on microbial composition, metabolite production, and downstream immune signaling. Although direct mechanistic studies in melanoma remain limited, a growing body of evidence suggests that fiber-based dietary interventions can shape antitumor immunity through many of the established immune pathways by which melanoma pathogenesis is controlled [7,36].

The proliferation or diminishment of microbial populations is largely shaped by dietary fiber, resulting in downstream alterations in antigen presentation, cytokine signaling, and T-cell priming [37]. Broadly, fiber-rich environments promote microbial diversity and enrich taxa that support immune homeostasis, whereas fiber depletion can shift communities toward mucin-degrading species that impair barrier integrity and heighten inflammation [20]. In a randomized trial of patients with type II diabetes, participants assigned to a high-fiber diet showed significant changes in their gut microbial diversity after three months compared to participants receiving standard care [38]. However, most of these shifts returned toward baseline microbial diversity after one year. These findings suggest that short-term dietary fiber interventions can rapidly reshape the GM, but long-term structural stability requires sustained dietary adherence. Even so, a modest increase in microbial richness persisted beyond the intervention period, indicating that certain microbial features may remain durably influenced by fiber even as the broader community structure returns toward baseline [38]. Notably, this broad microbial enrichment effect by dietary fiber persists in melanoma patients [19].

Soluble fibers are fermented by these bacteria into SCFAs, including acetate, propionate, and butyrate, which then exert systemic effects through both receptor-mediated and epigenetic mechanisms. SCFAs bind GPCRs throughout the body, such as GPR41, GPR43, and GPR109A, stimulating signaling cascades and promoting downstream immune cell migration, neutrophil activity, and regulatory T-cell development (Table 1) [25]. Clinical studies further support the relevance of SCFAs in melanoma. In patients receiving anti-PD-1 therapy, higher serum levels of acetic, butyric, propionic, and valeric acids were each associated with significantly improved PFS (*p* = <0.001, <0.001, <0.001, and 0.04, respectively) [39].

A substantially investigated example of SCFA immunomodulatory mechanisms, butyrate exerts its influence by promoting histone acetylation and tumor-suppressive gene expression via HDAC inhibition [31]. Additionally, butyrate enhances preservation of stem-like CD127^+^CD8^+^ T cells that are critical for sustained anti-tumor immunity in tumor-draining lymph nodes [40]. However, not all butyrate-mediated effects are uniformly beneficial. In human melanoma cells, butyrate has been shown to upregulate annexin A1, or ANXA1, leading to reduced E-cadherin expression and increased epithelial-to-mesenchymal (EMT) related invasion, suggesting that butyrate can also activate pro-invasive pathways under certain conditions [41]. Although this pathway has best been characterized in GI cancers, it provides a strong mechanistic rationale for how fiber-derived metabolites may shape melanoma immunity. Collectively, these findings suggest that SCFA availability may influence the strength or durability of antitumor T-cell responses during immunotherapy. Overall, fiber fermentation leading to SCFA-mediated GPCR signaling, increased expression of tumor-suppressor genes, and effects on DC and T-cell function represent mechanisms linking dietary fiber to immune modulation in melanoma.

#### 3.2.2. Dietary Patterns as Nonspecific Immune Modulators

High fiber diets, such as the Mediterranean diet, provide broader, albeit nonspecific, evidence for how fiber shapes the microbiome. The Mediterranean diet consists of high-fiber and low-red meat intake and is associated with greater gut microbial diversity compared with the typical Western diet, which is high in animal fat and protein but low in fiber [42]. The Mediterranean diet is associated with greater microbial diversity, increased abundance of SCFA-producing organisms, and improved markers of immune regulation [42]. In melanoma patients receiving immune checkpoint blockade (ICB), adherence to a Mediterranean-like diet has been associated with improved clinical outcomes, possibly reflecting increased SCFA production [43]. Plant-based diets also promote fiber-driven shifts, with foods such as cabbage, green leafy vegetables, and mushrooms contributing to higher SCFA levels [39]. More frequent mushroom intake is also linked with longer PFS [39].

Another study evaluating dietary patterns in melanoma patients found that high plant consumption, based on fruit and vegetable intake, was significantly associated with treatment response (*p* < 0.05) and was linked to a 2.9-fold higher likelihood of responsiveness to anti-programmed cell death protein 1 (PD-1) therapy [17]. However, when fruits and vegetables were evaluated separately, neither showed an independent association (*p* > 0.05), suggesting that the combined plant pattern may better capture relevant dietary components like fiber, folate, vitamins, and immune-active phytochemicals [17]. However, because these dietary habits were assessed before cancer treatment rather than during immunotherapy, the timing and relevance of these associations remain uncertain, and the exact mechanisms in these studies likely involve multiple dietary components rather than fiber alone. Taken together, these findings suggest that fiber may exert its effects within broader dietary patterns and in combination with immunotherapy, rather than as a single intervention.

Although some studies have reported lower melanoma risk among individuals with higher fruit and vegetable intake, including reductions ranging from roughly 34–46% for fruit and 40–57% for vegetables, these findings have not been consistent [44]. Notably, the largest cohort in this group did not observe any association, and the overall protective pattern appeared to be driven primarily by smaller studies [44]. Further investigation is needed to clarify this observation, evaluating if and how broad dietary patterns may confer repeatable, generalizable benefits for melanoma patients.

Controlled fiber deficiency models also emphasize the importance of fiber-derived metabolites in maintaining immune balance. When mice are deprived of dietary fiber, gut bacteria shift toward consuming host mucosal glycoproteins, leading to erosion of the colonic mucosal barrier [32]. In this setting, mice are more susceptible to lethal colitis from *Citrobacter rodentium* due to enhanced epithelial access [32]. These findings reflect how fiber inadequacy or deficiency can drive impaired microbial metabolism, reduced SCFA availability, and increased inflammatory susceptibility. These conditions may indirectly impair host immune responsiveness necessary for halting melanoma progression and responding to targeted therapeutics.

#### 3.2.3. Microbial Taxa and Metabolites Linking Fiber and ICI Response

Emerging mechanistic studies provide direct evidence that high-fiber diets can reprogram the TME in ways that support antitumor immunity and improve ICI efficacy. For example, butyrate-mediated enhanced proliferation of stem-like CD8^+^ T cells directly enhances ICI efficacy in melanoma by maintaining a reservoir of tumor-specific T cells capable of long-term immune surveillance [40]. Additively, pentanoate and butyrate treatment significantly enhanced CD8^+^ T cell antitumor activity [45]. This mechanism was mediated by metabolic and epigenetic reprogramming, including activation of mTOR signaling and inhibition of class I HDACs [45].

Network analysis identified *Akkermansia muciniphila* as a key microbial driver of the high-fiber phenotype, and monocolonizing germ-free mice with *Akkermansia* increased intratumoral DCs, shifted macrophages toward more immunostimulatory states, and improved tumor control. Akkersmansia metabolizes inulin, a dietary fiber, and has also been associated with increased immunotherapy response in metastatic renal cell carcinoma (mRCC) and metastatic non-small cell lung cancer (NSCLC) [46,47]. Studies also show that microbial communities enriched with *A. muciniphila* can restore or enhance PD-1 blockade activity, reinforcing its role as a beneficial bacterium in shaping immunotherapy response [48]. In cancer patients, oral *A. muciniphila* restored PD-1 sensitivity through an IL-12-dependent pathway and increased the recruitment of CCR9^+^CXCR3^+^CD4^+^ T cells into the tumor [9,48].

Evidence surrounding some microbial taxa is mixed. Compared with *A. muciniphila*, colonization with *Lactobacillus reuteri* produced the opposite pattern, reinforcing that not all bacteria contribute equally to antitumor immunity [9]. However, in another study, *Lactobacillus* reduced melanoma incidence in mice from seventy to thirty percent, and was associated with increased anti-cancer cytokines, and reduced melanoma tumor cell migration [49]. In preclinical melanoma models, *Lactobacillus reuteri* was shown to translocate into tumors and remain present, where it released the tryptophan-derived AhR (aryl hydrocarbon receptor) agonist indole-3-aldehyde [50]. This metabolite activated CD8^+^ T-cell-specific AhR signaling, increased IFN-γ production through cAMP response element-binding protein (CREB)-dependent pathways, and ultimately enhanced the antitumor activity of ICIs [50].

Many bacterial species, such as *Faecalibacterium*, *Enterococcus hirae*, and *Bacteroides fragilis,* have been previously linked to favorable ICI outcomes in humans, and are also capable of metabolizing pectin, suggesting a biologically plausible synergy between specific fiber types and beneficial microbial communities [9,19]. Although these experiments were performed in a lymphoma model, the immune pathways involved, especially STING–IFN-I signaling and DC recruitment, are also central to antitumor responses in melanoma, suggesting that the findings may have relevance beyond the original model.

Across models, dietary fiber and fiber-responsive microbes are associated with increased DC activation, stronger Th1 skewed T cell responses, and enhanced IFN-γ signaling, which may help support responses to ICIs.

#### 3.2.4. Fiber-Specific Immunomodulation

Different fibers selectively enrich distinct microbial taxa, and several of these taxa have been linked to favorable antitumor immunity in melanoma. Across studies, specific fermentable fibers, such as β-glucan, inulin, and pectin, appear to be the most consistently associated with immunomodulatory effects relevant to melanoma, mainly through microbiome-dependent mechanisms.

One study by Alexander et al. showed that fermentable fibers, namely β-glucans, shape antitumor immunity. Utilizing murine melanoma models, supplementation with *S. cerevisiae*-derived β-glucan reduces pulmonary melanoma metastases [51]. These effects appear to be partially driven by enhanced activation of monocytes and NK cells, suggesting that β-glucans help prime innate immune responses that prevent tumor spread. Given that the melanoma TME is often highly immunosuppressive, these innate-boosting effects may help counteract barriers to effective T-cell infiltration.

Inulin, a well-studied prebiotic fiber, stimulates the growth of *Bifidobacterium* and *Faecalibacterium* species, both associated with improved ICI responses in melanoma [16,19]. Inulin boosts IFN-γ-driven Th1 immunity from both conventional and γδ T cells, leading to deeper immune infiltration and better tumor control, while both bacterial species support antitumor immunity by stimulating DC activation, increasing major histocompatibility complex (MHC) expression, and enhancing Th1-polarized T-cell responses [52]. These findings highlight how specific fibers can promote antitumor immunity through microbial alteration of metabolites.

Several melanoma mouse models show that inulin can reinforce antitumor immune responses. Animals receiving inulin demonstrate slower melanoma progression and increased T-cell infiltration in the TME. These effects stem from enhanced DC activity, as inulin exposure promotes increased expression of MHC class I and II, making it easier for both CD4^+^ and CD8^+^ T cells to recognize and target tumor cells [53]. Notably, inulin alone triggered IFN-γ production in γδ T cells and CD4^+^ T cells to a degree comparable to anti-PD-1 therapy, highlighting its ability to independently activate multiple T-cell subsets essential for tumor control. Inulin combined with anti-PD-1 therapy also increased CD8^+^ T cell frequency compared with anti-PD-1 alone (*p* = 0.04), significantly slowing tumor growth [54]. Inulin was also associated with increased quantities of beneficial microbes, such as *Akkermansia*, *Lactobacillus*, *Roseburia*, and short-chain fatty acid (SCFA) metabolites [54].

Additionally, in EL4 murine lymphoma models, a high-fiber dietary intervention with pectin increased type I IFN signaling within tumors, boosted overall DC infiltration, and expanded cDC1 populations, immune cells that are critical for T-cell priming [9]. These changes were accompanied by slower tumor growth, whereas mice given a Western-style diet high in fat and sugar showed fewer intratumoral DCs and accelerated tumor progression [9]. Manipulating the microbiome with a narrow-spectrum antibiotic produced a similar reduction in DC recruitment, underscoring the GM’s role in shaping these effects.

Collectively, these findings highlight the mechanisms by which dietary fiber primes the immune system and modulates the dynamic between the host antitumor response and the melanoma TME.

#### 3.2.5. Summarized Impact of Fiber Metabolites and Fiber-Responsive Taxa on the Melanoma Tumor Microenvironment

The collective downstream impact of dietary fiber, namely microbial proliferation and fermentation producing SCFAs, on the melanoma TME are best characterized by stimulation of immune cells and supporting the intestinal barrier. SCFAs, such as butyrate, propionate, and acetate, collectively enhance CD8^+^ T cell infiltration into melanoma tumors while initiating signaling cascades driving gene expression modulation within the melanoma TME [19]. Beneficial bacteria like *Bifidobacterium* and *Ruminococcus*, enriched by high fiber intake, maintain the quality of microbiota-mediated colonic mucus, creating a protective barrier between gut microbiome and intestinal epithelium [14].

Melanoma is a UV-induced, non-mucosal tumor with a high tumor mutational burden and high baseline T-cell infiltration and immunogenicity [55]. The melanoma TME is also characterized by immunosuppressive myeloid cell, such as myeloid-derived suppressor cells, and tumor-associated macrophage predominance, which can impair antitumor responses [56]. Alternative tumor models implicated for fiber-based investigations, such as GI and lymphoma, exhibit entirely different immune composition, stromal architecture, and mechanisms of immune regulation, consequent of their pathogenic drivers [57]. Further, melanoma does not develop with direct contact with the gut microbiome, and the immunomodulatory effects of SCFAs likely work through indirect rather than local effects [19].

Considering the complex differences in TME between these tumor models, the impact of dietary fiber intervention likely varies significantly, and context-specific interpretation is key for understanding. Inferring from the differing characteristics of the melanoma TME, the impact of dietary fiber and SCFAs likely varies compared with other cancer models. In tumors that exhibit lesser baseline immunogenicity or mucosal origin, dietary fiber and its metabolites likely play a larger role in immune activation and antitumor responses. In melanoma, where immune cells are largely present but suppressed by tumor-associated myeloid cells, fiber may have more subtle, indirect, and context-dependent effects [58]. Rather than directly initiating immune responses or affecting immunosuppressive cells, fiber and its metabolites may increase existing T cell infiltration or activation within the tumor. Overall, the influence of fiber on the melanoma TME is likely characterized by influencing existing immune responses, highlighting the importance of interpreting pathophysiological responses to interventions specific to the cancer type.

#### 3.2.6. Overview of Preclinical Studies

Preclinical studies utilizing melanoma models have provided important insight into the mechanisms driving antitumor immunity by fiber-responsive microbial taxa. Although some mechanistic studies come from non-melanoma models, many of the pathways involved, including DC activation, Th1 polarization, and type I IFN signaling, are central to melanoma biology and offer relevant clues for how fiber-driven microbial changes may reshape immune responses [9,53]. Many relevant preclinical findings have been mentioned throughout this section. Taken together, current preclinical studies suggest that dietary fiber and fiber-responsive taxa can remodel the tumor immune microenvironment and influence checkpoint blockade activity (Table 2).

This table summarizes preclinical studies evaluating how dietary fiber, microbial metabolites, or gut commensal supplementation modulate antitumor immunity in melanoma-bearing mice. Across models, interventions such as inulin, pectin, yeast-derived β-glucan, and live *Bifidobacterium* directly influenced DC activation, T-cell priming, macrophage polarization, NK cell recruitment, and cytokine signaling pathways that shape the TME. Mechanistic findings highlight both microbiota-dependent and microbiota-independent effects, revealing how distinct fiber types and microbial taxa alter immune infiltration, IFN-γ-driven Th1 responses, γδ T-cell activation, and sensitivity to immune checkpoint blockade. Collectively, these studies illustrate the complex interactions between diet, microbial composition, and host antitumor immunity in melanoma models.

Preclinical findings have motivated ongoing clinical efforts to investigate the similarity of these patterns and immunomodulatory effects in melanoma patients.

### 3.3. Clinical Studies and Observational Data in Humans

#### 3.3.1. Fiber Intake and Melanoma Risk

The average daily fiber intake in the United States is approximately 16 g, and comes mainly from cereals, fruits, and vegetables [59]. The Institute of Medicine recommends 19–38 g per day with variation based on age and gender [60]. Higher fiber intake often reflects an overall nutrient-dense diet rich in fruits, vegetables, legumes, and whole grains.

#### 3.3.2. Observational Data

A large, prospective cohort study found that fiber intake was consistently protective against several cancer types, including colorectal, lung, esophageal, and kidney cancers. Notably, these associations did not extend to melanoma, as United Kingdom (UK) Biobank data from approximately 190,000 individuals showed no significant relationship between fiber consumption and melanoma incidence [61].

One explanation for this unexpected finding is that melanoma may be less sensitive to the pathways through which fiber typically exerts its benefits. SCFAs tend to act on epithelial surfaces, modulate local inflammation, and influence carcinogenesis in the GI tract and nearby organs [25]. Melanoma, which is more strongly driven by ultraviolet (UV) exposure, pigment biology, and immune surveillance rather than mucosal inflammation, may simply not rely as heavily on these fiber-responsive mechanisms. Additionally, the beneficial impact of dietary fiber against melanoma may be more relevant during treatment, particularly as an adjuvant to ICIs, rather than as a monotherapy given especially in the early stages of tumor progression.

Further, although the UK Biobank study was thorough and novel, there were significant limitations reported. Confounding cannot be fully ruled out, and dietary fiber intake was self-reported, introducing recall bias that could weaken true associations. The use of 24 h dietary recalls also limits the ability to capture long-term habitual intake, and participants’ diets may have changed over time without being reassessed. Additionally, the cohort involved lacked representativeness in relation to the general population; participants tended to be healthier, more health-conscious, and predominantly of British-white ethnicity, which may limit generalizability to more diverse melanoma populations. Other unmeasured factors, such as sun exposure patterns or pigment-related risk modifiers, may also have influenced the results [61].

In a different study, obesity, which is typically associated with lower fiber intake, was associated with improved PFS and overall survival (OS) in metastatic melanoma with the strongest effect seen in men receiving anti-PD-1 therapy [62]. This unexpected “obesity paradox” highlights how nutrition and metabolism can interact with melanoma biology in complex ways and reinforces the need to consider dietary factors alongside other variables that influence treatment response. Still, these findings establish that dietary fiber take plays some immunomodulatory role relevant to other non-GI tract cancers, supporting continued investigation into its broader immunologic effects [63].

Various hypotheses may help explain how both high fiber intake and obesity have been associated with improved immunotherapy outcomes in different contexts. One hypothesis is that obesity alters metabolism in ways that may affect immune cell function, such as changes in cytokine signaling that may support immune activation during immune checkpoint blockade in certain patients. Alternatively, these findings may suggest that diet and body composition influence antitumor immunity through partially independent pathways. High fiber intake may work primarily through microbiome-mediated mechanisms, such as increased SCFA production, and enhanced DC and T-cell activation.

In contrast, obesity’s effects may work more through host metabolic or hormonal factors rather than direct changes to the microbiome or fiber-related mechanisms. Rather than representing opposing variables, fiber intake and obesity reflect distinct biological influences that shape immune responsiveness in melanoma in different contexts. Dietary fiber may have limited relevance for melanoma incidence or prevention, but plays a more meaningful role in shaping immune responses during melanoma immunotherapy.

Together, these observations suggest that diet, metabolic state, and the gut microbiome interact in complex ways in melanoma, and that no single measure is likely to fully explain differences in response to immunotherapy.

#### 3.3.3. Fiber, the Microbiome, and Response to Immune Checkpoint Inhibitors in Clinical Studies

Immune checkpoints, such as PD-1/programmed death-ligand 1 (PD-L1) and cytotoxic T lymphocyte-associated protein 4 (CTLA-4), are cell-surface molecules that play a regulatory role in the progression or suppression of immune responses [2]. In cancer immunology, immune checkpoint inhibitors block inhibitory checkpoint signaling often through antibody-mediated mechanisms, enabling T-cell activation and subsequent enhancement of antitumor responses [2]. Nivolumab, an anti-PD-1 therapy, and ipilimumab, an anti-CTLA-4 therapy, have both been shown to improve survival in patients with advanced melanoma [64]. Atezolizumab in combination with cobimetinib and vemurafenib has also been approved for use in B-Raf proto-oncogene (BRAF)-V600 mutation-positive unresectable or metastatic melanoma [2].

Although effective as melanoma therapeutics, ICIs are limited as monotherapies by variable response rates, tumor-type selectivity, and severe irAEs [65]. To combat this, combination immunotherapy has become an important strategy in melanoma treatment. For instance, some studies have demonstrated that anti-PD-1 and anti-Cytotoxic T lymphocyte-associated protein 4 (anti-CTLA4) combination therapy results in higher response rates and increased PFS and OS rates [66]. Thus, future efforts aim to elucidate the factors driving optimal and adverse patient-therapeutic matching to maximize treatment responsiveness, minimize the incidence of irAEs, and advise combination therapy strategies [2].

ICI response is influenced by a wide range of tumor and host factors, including tumor mutational burden (TMB), IFN signaling, quantity and phenotype of tumor-infiltrating immune cells, epigenetic changes, intestinal microbiome, and HLA type [65]. Within this context, the GM has emerged as a modifiable host factor that may influence treatment efficacy and response. Given that diet shapes microbial communities and composition, current efforts aim to explore the ability of targeted dietary changes, such as increased dietary fiber and prebiotics, to support better outcomes with existing immunotherapy.

Recent studies have demonstrated the impact of increased intake of fermentable fibers, such as inulin, on enrichment of *Faecalibacterium* and *Bifidobacterium*, two taxa consistently associated with improved PD-1/PD-L1 responses [2]. In murine melanoma models, enriching the gut with *Bifidobacterium* markedly improves tumor control and enhances antitumor T-cell activity, including increased CD8^+^ infiltration and IFN-γ production [50,67]. These effects rely on live bacteria and correlate with increased DC activation, highlighting the ability of specific commensal species to strengthen antitumor immunity independently of bacterial translocation [50]. Similar patterns have been found with anti-CTLA4 blockade. In preclinical melanoma models, germ-free or antibiotic-treated mice showed a reduced response to anti-CTLA4 therapy, yet therapy sensitivity could be restored after reintroducing Bacteroides fragilis species into the gut [68].

Human studies also support these findings. Patients enriched for *B. longum*, *E. faecium*, and *C. aerofaciens* demonstrate stronger systemic T-cell activation and more favorable PD-1 inhibitor responses [12]. Additionally, patients enriched for *Clostridiales*, *Ruminococcaceae*, and *Faecalibacterium* show higher baseline densities of CD4^+^ and CD8^+^ T cells, and stool from responders drives a similar immune profile in mice, with increased CD8^+^ T cells and reduced suppressive myeloid and regulatory T-cell populations [16]. Responders additionally had higher alpha diversity (*p* < 0.01), greater abundance of Ruminococcaceae (*p* < 0.01), and enrichment of anabolic pathways like amino acid biosynthesis, suggesting that both community structure and microbial function contribute to improved ICI outcomes [16]. These findings suggest that microbial composition, along with microbe-derived antigens and metabolites, may serve as useful biomarkers of response to immune checkpoint blockade in melanoma [69].

Furthermore, recent work has led to the development of a predictive topological biomarker score called TOPOSCORE, which predicts ICI responsiveness based on patterns of gut dysbiosis, quantified in part by the presence of *Akkermansia* species. This score has been validated in advanced cutaneous melanoma. The identified microbial species most frequently associated with ICI responders were *Akkermansia muciniphila, Bifidobacterium pseudocatenulatum*, and *Roseburia* spp. However, no one species was found in every responder, suggesting that ICI response may rely more on the overall diversity of the microbiome, rather than the presence of specific species alone [70].

Another study by Spencer et al. consisting of melanoma patients (*n* = 128) undergoing anti-PD-1 therapy found that patients who reported increased dietary fiber (>20 g/day) were associated with an improved PFS (*p* = 0.047) [19]. Moreover, each 5 g increase in daily fiber consumption resulted in a 30% decrease in the risk of disease progression or mortality [19]. Patients with the highest fiber intake also experienced slower disease progression and were more likely to respond to treatment, even after accounting for other clinical factors and recent antibiotic use [19]. Interestingly, the patient group receiving dietary fiber supplementation without addition of probiotics received the greatest benefit from the intervention to their overall outcomes [19]. These findings support the hypothesis that regular and adequate fiber intake may help create a GM that supports more effective ICI activity. Although these findings come primarily from studies in non-melanoma solid cancers, antibiotic exposure has repeatedly been associated with reduced microbial diversity, loss of beneficial taxa such as *Ruminococcus*, and increased representation of immunosuppressive or proinflammatory organisms, including *Enterocloster* and *Hungatella* [71]. These patterns may be relevant to melanoma as well, given the shared reliance on intact gut microbial ecosystems for effective ICI activity.

Additional prospective data also suggest that both diet and native microbial profiles may influence how patients respond to immunotherapy. In a multicenter cohort of patients from Australia, the Netherlands, and the United States, receiving neoadjuvant ICIs for high-risk melanoma, distinct microbial communities were associated with treatment response across different geographic regions. Dutch patients were more likely to have *Ruminococcaceae*-dominant microbiomes, US patients were more likely to have *Bacteroidaceae*-dominant microbiomes, and Australian patients showed a mix of both. These microbial patterns became highly relevant, as individuals with *Ruminococcaceae*-enriched communities experienced higher response rates compared with those dominated by *Bacteroidaceae* [18]. Lower intake of dietary fiber and omega-3 fatty acids, along with higher baseline C-reactive protein levels, were more commonly seen in nonresponders [18]. These findings highlight how habitual diet, systemic inflammation, and underlying microbial ecology may work together to shape ICI efficacy and toxicity.

A recent review of nineteen studies further supports the relationship between diet and immunotherapy response. Across six prospective human cohorts, higher fiber intake and adherence to Mediterranean-style eating patterns were consistently linked to better outcomes with a pooled odds ratio of 5.79 favoring fiber-rich diets [72]. These dietary patterns also aligned with higher levels of SCFAs and lactic acid-producing bacteria such as *Faecalibacterium prausnitzii* and *Akkermansia muciniphila*, which correlated with improved objective response rates. Mouse studies mirrored these findings, showing that fiber-enhanced diets increased SCFA production and strengthened anti-PD-1 activity, while fasting or fasting-mimicking diets reduced tumor volume [72]. Notably, long-term survivors in several human studies consumed less sugar despite similar macronutrient intake overall, suggesting that low-sugar, fiber-rich patterns may complement immunotherapy [72]. Taken together, these data indicate that fiber-rich diets, or related changes, such as Mediterranean diet adherence, increased SCFA levels, and beneficial bacterial shifts are consistently associated with improved ICI efficacy across both clinical and preclinical settings.

#### 3.3.4. Microbiome and ICI Toxicity

The recent literature has suggested that certain gut microbial profiles may also reduce ICI-related toxicities, although this association requires further validation. The GM not only affects how patients respond to ICIs but also determines who develops treatment-related toxicities. One of the most commonly reported irAEs is immunotherapy-induced colitis, which is suspected to result from the loss of normal self-tolerance and increased T-cell activity [73]. In one study by Chaput et al., patients with higher levels of *Faecalibacterium* were more likely to develop colitis during anti-CTLA4 therapy, while those with greater amounts of Bacteroides were less affected [74]. Interestingly, the group enriched with *Faecalibacterium* had greater responses to treatment (associated with CD4 T cells), whereas Bacteroides dominant individuals experienced fewer toxicity events but saw less clinical benefit. In combination therapy, similar results were found, where Bacteroides species were associated with lower colitis risk [75]. Overall, these results highlight the delicate balance between efficacy and safety in immunotherapy.

In addition to microbial patterns, early clinical evidence also suggests that dietary fiber may influence the risk of GI toxicities during ICI treatment. In a small cohort of patients receiving a ipilimumab–nivolumab combination for advanced melanoma, those who were at lower risk of immune-related colitis tended to consume more fiber relative to their total energy intake and were closer to recommended dietary levels [76]. Other macronutrient intake and overall caloric consumption did not differ meaningfully between groups. Higher fiber intake was associated with less diarrhea and colitis, raising the possibility that dietary fiber may help modulate irAE risk in the setting of combination checkpoint blockade. Fiber’s effects in reducing colitis risk and severity has also been shown in ulcerative colitis, with reduced relapses, rates of gut dysbiosis, and inflammatory markers [77].

Given the microbiome’s influence on both response and toxicity, especially colitis, there is increasing interest in whether dietary fiber can be used to modify this risk-balance. Several clinical trials are now examining fiber-based interventions in melanoma patients receiving ICIs.

#### 3.3.5. Ongoing Clinical Trials

Given the growing evidence linking gut microbiota composition with immunotherapy efficacy, clinical research efforts have shifted from observational associations to prospective dietary and GM-modulating interventions in melanoma patient cohorts. These trials aim to assess whether fiber can improve treatment response, reduce toxicity, or influence microbiome composition in melanoma patients. An early case report described two patients with metastatic melanoma whose disease progressed despite ICI therapy with significant irAEs, but showed significant treatment responsiveness when rechallenged with a combination therapy of nivolumab and the prebiotic, camu camu [78]. Both patients achieved near-complete or complete responses shortly after adding camu camu, experiencing disease control for over a year and minimal recurrence of irAEs [78]. Although limited to two cases, these findings raised the possibility that camu camu-mediated microbiome modulation may enhance ICI efficacy and tolerability, providing the rationale for the now ongoing clinical trial and further investigation evaluating camu camu in melanoma patients (Table 3).

This table summarizes active interventional clinical trials evaluating how dietary manipulation, prebiotic supplementation, or combined lifestyle interventions influence gut microbiome composition, systemic immune activation, and treatment outcomes in melanoma patients receiving immune checkpoint inhibitors. Trials include diet-based interventions such as prebiotic food-enriched diets, high-fiber supplementation, fermented foods, structured diet counseling, and exercise programs. Reported data include trial number, enrolled population, intervention, and primary outcome measures, reflecting ongoing efforts to understand how targeted modulation of the gut microbiome may enhance antitumor immunity or improve immunotherapy response in melanoma.

Parallel with ongoing interventional efforts, several observational studies are investigating how naturally occurring variation in the GM may influence immunotherapy outcomes in melanoma (Table 4).

**Table 4:** Ongoing Observational Microbiome Trials in Melanoma and Immunotherapy.

Trial ID	Title	Population	Enrollment	Primary Outcome Measured
**NCT04107168** [88]	Microbiome Immunotherapy Toxicity and Response Evaluation	Unresectable stage 3 or 4 melanoma, advanced RCC, or NSCLC patients receiving anti-PD(L)1 +/− anti-CTLA-4 antibodies	1800 (est)	Ability to predict for PFS of 1 year or greater for patients with advanced melanoma, renal, and non-small cell lung cancer.
**NCT03643289** [89]	Predicting Response to Immunotherapy for Melanoma With Gut Microbiome and Metabolomics (PRIMM)	Stage 3 or 4 melanoma patients initiating first-line immunotherapy	450 (est)	GM diversity and peripheral blood mononuclear cells immunophenotyping in relation to responses to treatment and side effects in patients with stage 3 or stage 4 melanoma receiving immunotherapy.
**NCT05037825** [90]	The Gut Microbiome and Immune Checkpoint Inhibitor Therapy in Solid Tumors (PARADIGM)	Malignant melanoma, NSCLC, RCC, TNBC patients initiating anti-PD-1, anti-PD-L1, or anti-CTLA-4	800 (est)	Microbiome evaluation with whole metagenome shotgun sequencing to assess changes in the relative abundance of microbial taxa (measured as percentage abundance per microbial species and changes in percentage abundance between baseline and cycle 2 timepoints) in patients who are receiving checkpoint blockade immunotherapy as the standard of care.

This table outlines ongoing observational studies assessing the relationship between baseline gut microbiome profiles, microbial metabolites, immune signatures, and clinical outcomes in melanoma patients undergoing ICI therapy. These cohorts do not involve dietary or interventional exposures and aim to characterize natural microbiome variation and identify microbial or metabolomic biomarkers linked to treatment response, PFS, or irAEs. Data presented include population characteristics, planned enrollment, and primary outcome measures, highlighting current efforts to define microbiome-based predictors of immunotherapy efficacy and toxicity.

#### 3.3.6. Counterevidence and Methodological Challenges

While several studies suggest that dietary fiber may support better responses to ICIs, newer evidence shows that this relationship is not as consistent as originally thought.

A recent set of murine experiments (pre-print article) evaluated fiber’s impact on ICB across multiple tumor models and found that the effect of fiber was often weak, inconsistent, or dependent on factors unrelated to fiber itself [91]. Closer analysis of these findings revealed a major limitation with respect to murine diets. Grain-based chow, which is naturally high in fiber but also rich in other phytochemicals, micronutrients, and plant compounds, was used as a source of dietary fiber for comparison to low-fiber purified diets. The uncontrolled, likely unregulated presence of other nutrients in the chow substantially weakened the assertion that fiber alone was responsible for the observed benefits [91]. A follow-up comparison was conducted comparing chow with both high-fiber and low-fiber purified diets, finding that the “metabolome” of chow-fed mice showed minimal overlap compared with both purified diets [91]. Thus, the differences in resulting ICB activity were more attributed to the presence of other nutrients in the chow rather than the fiber content. This distinction is important when reviewing preclinical studies summarized in Table 2, where grain-based chow is compared to fiber-altered diets, since observed immune or antitumor effects cannot be solely attributed to fiber itself. The findings described in Table 2 may reflect overall effects of broad diet changes leading to differences in the gut microbiome or immune response.

Other limitations to standardizability arise among behaviors of different melanoma models: YUMM1.1-9 tumors responded more favorably to cellulose-rich purified diets, while B16-OVA tumors showed no diet-related or fiber-related differences in ICB response [91]. Similar variability appeared in a breast cancer model, where high-fiber purified diets modestly slowed tumor progression with anti-PD-1 therapy but did not improve survival or lung metastases.

This variability across different tumor models likely reflects underlying biological differences rather than inconsistencies across experiments. Melanoma models have varying levels of immunogenicity and immune cell infiltration, which may influence the responsiveness to dietary fiber or microbiome changes. Models that are more likely to respond to changes in fiber or the microbiome are those with greater immunogenicity at baseline and preserved antigen presentation. In subcutaneous melanoma models where the gut microbiome remained intact, fiber interventions were associated with increased DC activation, enhanced antigen presentation and increased CD4+ and CD8^+^ T cell infiltration [51]. Furthermore, commensal supplementation studies in melanoma show that live bacteria can enhance DC activation and CD8^+^ T cell priming, resulting in overall improved tumor control and response to immunotherapy [54].

In contrast, melanoma models that are less likely to respond are those that are less immunogenic at baseline. For example, B-16 melanoma models have limited T cell infiltration, weaker antigen presentation, and are less likely to engage adaptive immune responses due to deficient MHC Class I expression [92]. In these settings, dietary fiber alone may have a limited function to generate a de novo or enhanced antitumor response since there is minimal pre-existing T cell activity at baseline. This explains why, within melanoma, different models can respond differently. For example, in the same study, YUMM 1.1-9 tumors had a greater responsiveness to a cellulose-rich purified diet, while B16-OVA tumors had no diet or fiber-related differences in ICB response [91]. These differences in response to fiber are likely due to underlying differences in the baseline immune function of the models used.

Tumor implantation site can also contribute to variability among models. Subcutaneous melanoma models are often more accessible and easier for immune cells to infiltrate, which allows for easier assessments of immune cell infiltration. Many of fiber and microbiome modulation’s most significant effects in melanoma were reported in these settings, such as increased DC recruitment, greater T-cell responses, and enhanced IFN-γ signaling. On the other hand, metastatic models may rely more on innate immune mechanisms with fewer adaptive immunity mechanisms. For example, in a pulmonary metastatic melanoma model, β-glucan reduced metastatic burden through inflammatory monocyte-dependent pathways that were not reliant on adaptive immunity [51]. This suggests a setting where innate immunity predominates over T-cell-mediated mechanisms.

Altogether, these findings suggest that dietary fiber and microbiome changes are most likely to enhance antitumor effects in melanoma models that have greater antigen presentation and T-cell infiltration at baseline. Conversely, poorly immunogenic models with limited immune engagement or metastatic models more reliant on innate immune mechanisms may be less likely to respond to fiber alone and require combination strategies to achieve a measurable benefit or response. Across all models, the overall pattern did not support the idea that higher fiber reliably enhances ICB efficacy. This suggests that fiber’s effects on tumors and immunotherapy are context-dependent and shaped by tumor-specific characteristics such as tumor immunogenicity and dominant immune pathways. Fiber’s effects may also depend on broader dietary patterns, which emphasizes the need to interpret existing and prospective dietary fiber studies with caution and consider the complexity of whole diets and their components, along with the specific models utilized.

#### 3.3.7. Adherence, Tolerability, and Barriers to Fiber Intake

Achieving adequate fiber intake can be challenging, and willingness to modify diet varies substantially among patients. Common side effects of increased dietary fiber intake include bloating, abdominal discomfort, excessive gas, and alterations in bowel frequency [93]. In one randomized controlled trial, 36% of eligible participants declined enrollment specifically due to reluctance to change dietary habits [94]. Another study reported that individuals who experienced fiber-related GI symptoms were more likely to reduce their intake or withdraw from the trial altogether [94]. These observations suggest that even if fiber demonstrates clinical benefit in melanoma, real-world implementation may be constrained by issues of tolerability and adherence. Nevertheless, dietary modification in oncology populations has generally been shown to be safe and feasible, particularly when guided by structured dietary patterns, such as the Mediterranean diet [95]. It is also important to recognize that fiber’s effects may be influenced by an individual’s baseline microbiome composition and that no standardized dosing recommendations for prebiotics or dietary fiber currently exist in oncology guidelines.

### 3.4. Future Directions

#### 3.4.1. Emerging Areas of Research

Although dietary fiber modulates the GM primarily through fermentation and SCFA production, more direct microbiome-modifying approaches are also gaining interest. Early melanoma studies indicate that transferring the GM of ICI responders through fecal microbiota transplant (FMT) may help restore treatment sensitivity in a subset of nonresponders, and, in some cases, induce responsiveness in previously refractory tumors [96,97]. Multiple ongoing clinical trials are evaluating the combined effects of ICIs and FMT [98,99]. While FMT lies outside the scope of this fiber-focused review, results from these studies will further describe how deliberate microbiome restructuring can influence immunotherapeutic outcomes.

In parallel, other research is investigating how additional dietary components, including red meat, caffeine, and alcohol, may shape melanoma pathogenesis or therapeutic response [100]. As evidence accumulates linking fiber, the GM, and ICI response, specific microbial features may eventually serve as predictive biomarkers for treatment efficacy or toxicity, especially if reproducible microbial signatures distinguishing responders and nonresponders continue to emerge.

#### 3.4.2. Clinical Considerations for Fiber-Based Interventions

Responses to microbiome modulation through dietary fiber are unlikely to be uniform across patients. Individual factors, such as age, host genetics, immune status, baseline microbial diversity, and baseline gut barrier integrity, may all influence the degree to which fiber alters the microbiome or augments immunotherapy response. These considerations suggest that future strategies will likely require personalized dietary or prebiotic interventions rather than a universal approach.

Advances in artificial intelligence and machine learning offer promising avenues for this personalization. Emerging analytical frameworks can integrate multidimensional data from fecal microbiome profiling to predict treatment responsiveness, stratify patients, and potentially guide therapeutic decisions [2]. Such tools may ultimately enable individualized dietary recommendations or targeted microbiome modulation strategies tailored to a patient’s unique microbial and immunologic landscape.

#### 3.4.3. Dietary Fiber as an Adjunct to Immunotherapy

Dietary modification is relatively inexpensive and safe compared with most melanoma therapies. However, adherence and tolerability may influence real-world effectiveness, as outlined above, and current evidence does not support dietary fiber as a stand-alone treatment. Instead, fiber may function best as an adjunct to existing therapies, particularly ICIs, where it could help support more favorable microbial, gut tissue, and immune environments. Further, while associations between specific fiber types and improved therapeutic responsiveness have been observed, prospective study designs using combinations of specific fibers with and without conventional therapeutics have not yet been thoroughly investigated in melanoma models.

#### 3.4.4. Recommendations for Future Research

Several clinical trials are investigating microbiome-modifying interventions in melanoma, but data remain limited (Table 3 and Table 4). Many studies combine dietary fiber interventions with standard-of-care immunotherapy, which requires careful comparison of the GM before, during, and after treatment through stool, serum, and tissue sampling. Understanding both short- and long-term effects of GM modulation will require study designs that include appropriate control arms to ensure interpretable results.

Once safety and tolerability are well established, future trials should prioritize objective clinical endpoints, such as overall response rate (ORR), OS, PFS, and quality of life. Because adherence to dietary interventions is highly variable and fiber intake is challenging to precisely quantify, additional research is needed to evaluate feasibility, long-term tolerability, and strategies to promote sustained dietary modification.

A major limitation of existing work is the inconsistency in dietary assessment methods and the lack of standardized definitions for high versus low fiber intake [101]. These discrepancies limit cross-study comparisons and obstruct efforts to synthesize the body of evidence. Standardized fiber assessment tools and intake categories will be essential for producing reliable, comparable, and clinically meaningful data in future investigations.

## 4. Conclusions

The impact of dietary fiber on the GM in melanoma remains underexplored, and distinguishing association from causation will be essential as research progresses. A deeper understanding of how the TME, the GM, and immunotherapy mechanisms interact is needed to clarify fiber’s role. Current evidence suggests that fibers such as β-glucan and inulin may influence antitumor immunity through both microbial enrichment and immune modulation, although findings across preclinical and clinical studies remain variable and context-dependent. Observational and early interventional data suggest that higher fiber intake is associated with more favorable responses to ICIs and may be associated with differences in survival and treatment-related toxicity. Overall, fiber should not be viewed as a therapeutic intervention on its own and instead represents a generally well-tolerated area for further investigation as a potential adjunct to melanoma immunotherapy.

## Figures and Tables

**Table 1:** Dietary fiber-derived short-chain fatty acids and their GPCR targets. Overview of major dietary fiber-derived short-chain fatty acids (SCFAs), their primary G-protein-coupled receptor (GPCR) targets, and reported functions relevant to immune signaling and carcinogenesis.

SCFA	GPCRs	Function(s)
Acetate	GPR43	Reduced or absent in metastatic colorectal tumors; restored GPR43 expression induces G0/G1 cell cycle arrest and apoptosis in adenocarcinoma cell lines [26,27]. GPR43 mediates SCFA signaling through Gi/o and Gq pathways [28].
Butyrate	GPR41 GPR109A	GPR109A is silenced in colorectal cancer via DNA methylation; reexpression of GPR109A in the presence of butyrate induces apoptosis and suppresses NF-KB signaling [29]. Butyrate can also signal through GPR41.
Propionate	GPR41 GPR43	GPR41 is primarily activated by propionate and inhibits cAMP signaling through Gi/o pathways [28,30].

**Table 2:** Preclinical Models Demonstrating Microbiota- and Fiber-Driven Modulation of Melanoma Immunity.

Author	Model	Intervention	Key Mechanistic Findings and Results
Alexander et al. [51]	C57BL/6J mice: pulmonary metastatic melanoma	Yeast-derived β-glucan particles (YGP) vs. phosphate-buffered saline (PBS)	β-glucan (YGP) enhanced antitumor activity by: • Upregulating inflammatory mediators TNFα, M-CSF and CCL2 • Increasing lung neutrophils and inflammatory monocytes • Reducing tyrosinase expression in metastatic foci • Exerting anti-tumor activity independent of adaptive immunity and NK cells but dependent on inflammatory monocytes • Augmenting monocyte cytotoxicity against B16 melanoma
Boucher et al. [52]	C57BL/6 mice: melanoma	7.2% Inulin in drinking water + intraperitoneal anti-PD-1 therapy	Inulin enhanced antitumor activity by: • Increasing *Bifidobacterium* abundance • Enhancing IFN-γ-driven Th1 immunity • Increasing CD4^+^ and CD8^+^ αβ T-cell infiltration • Promoting γδ TIL activation • Augmenting overall CD45^+^ immune infiltration -This effect required an intact gut microbiota, as antibiotics eliminated inulin-induced tumor control. -Inulin alone elicited tumor control comparable to anti–PD-1 therapy and activated CD8+ TILs to a similar degree.
Han et al. [54]	BALB/c mice: colorectal carcinoma C57BL/6 mice: melanoma	Oral inulin gel + intraperitoneal administration of anti-PD-1 therapy	Inulin enhanced antitumor activity by: • Increasing circulating CD8^+^ T cell frequency compared with anti-PD-1 alone • Slowing tumor growth • Increasing the abundance of key commensal microbes and SCFAs • Enhancing IFN-γ-producing cytotoxic T cells • Promoting stem-like TCF1^+^ PD-1^+^ CD8^+^ T cells associated with durable antitumor immunity
Lam et al. [9]	C57BL/6 mice: melanoma	30% pectin fiber diet vs. 45% kcal Western diet	Pectin fiber enhanced antitumor activity by: • Remodeling the gut microbiota to activate type I IFN signaling • Increasing Xcl1^+^ NK cells • Expanding intratumoral DCs, including cDC1s • Identifying *Akkermansia muciniphila* as the key mediator that restores intratumoral DCs, improves macrophage polarization and suppresses tumor growth -Showed that Western diet, vancomycin, and *Lactobacillus reuteri* disrupted immune responses and promoted tumor growth.
Li et al. [53]	C57BL/6J mice: melanoma	Mucin (3% in drinking water) or inulin (15% supplemented in chow) versus standard chow	Inulin enhanced antitumor activity by: • Increasing total CD45^+^ leukocytes infiltration • Expanding effector CD4^+^ and CD8^+^ T cells and DCs • Upregulating chemokines and antigen-presentation gene expression • Reducing melanoma resistance to MEK inhibition -Demonstrated that an intact gut microbiota is required for eliciting these immune-mediated antitumor effects.
Sivan et al. [50]	C57BL/6J mice: B16.SIY melanoma	Direct administration of live *Bifidobacterium*	*Bifidobacterium* enhanced antitumor immunity by: • Augmenting DC activation • Increasing CD8^+^ T-cell priming and infiltration • Increasing IFN-γ production -Nearly eliminated tumor outgrowth when combined with anti-PD-L1 therapy; required viable bacteria and intact CD8^+^ T cells.

**Table 3:** Ongoing Clinical Trials Investigating Dietary Fiber, Microbiome Modulation, and Melanoma Outcomes.

Trial ID	Title	Population	Enrollment	Intervention	Primary Outcome Measured
NCT05303493 [79]	Camu-Camu Prebiotic and Immune Checkpoint Inhibition in Patients With Non-small Cell Lung Cancer and Melanoma	Advanced melanoma and NSCLC patients	45 (act)	500 mg Camu Camu capsule + ICI	Incidence of treatment-related adverse events (safety and tolerability) in patients with NSCLC and melanoma.
NCT06475807 [80]	Dietary Interventions in Cancer Patients Treated With Immune Checkpoint Inhibitors	Melanoma or NSCLC patients + standard of care anti-PD-1/PD-L1 therapy	60 (est)	High-fermented foods and high-fiber supplements	Change in cell frequency of gut microbiota composition.
NCT04645680 [81]	Effect of Diet on the Immune System in Patients With Stage III-IV Melanoma Receiving Immunotherapy, DIET Study (DIET)	Stage III/IV melanoma or unresectable RCC patients starting pembrolizumab or nivolumab	50 (act)	Isocaloric high fiber diet vs. isocaloric control diet	Change in the GM.
NCT06298734 [82]	High-Intensity Exercise and High-Fiber Diet for Immunotherapy Outcomes in Melanoma Patients: The DUO Trial	Advanced melanoma patients receiving ICI therapy (anti-PD-1, anti-CTLA4, ± anti-LAG3 mAbs)	40 (est)	Group A: High intensity exercise (EX) Group B: High Fiber Diet (DT) Group C: Combined (COMB)	Change in GM diversity.
NCT06250335 [83]	Impact of a Prebiotic Food-enriched Diet (PreFED) in Combination With Ipilimumab/Nivolumab Combination ICB in ICB-refractory Melanoma Patients	Stage III/IV ICB refractory metastatic melanoma patients starting ipilimumab + nivolumab	4 (act)	Prebiotic Food-Enriched Diet (Pre-FED) PLUS Ipi or Nivolumab	Overall response rate (ORR).
NCT06236360 [84]	Metastatic Melanoma Patients on Immunotherapy With Nutritive Intervention Based on Mediterranean Diet (MINI-MD)	Metastatic melanoma patients receiving anti-PD-1 and anti-CTLA4	30 (est)	Mediterranean Diet	Change in the level of ingested flavones, fiber, anthocyanins, omega-3 fatty acids, and vitamin D.
NCT06548789 [85]	Neoadjuvant Immune Checkpoint Blockade + a Prebiotic Food-enriched Dietary Intervention to Optimize Immune Response in Melanoma: NEO-PreFED	Resectable melanoma patients starting neoadjuvant ipilimumab + nivolumab or nivolumab + relatlimab	35 (est)	Prebiotic food-enriched diet (PreFED) + neoadjuvant ICB	Completion rate, compliance, and adherence.
NCT06466434 [86]	Prebiotic Food-enriched Diet (PreFED) to Enhance the Microbiome and Response to First-line Immunotherapy in Unresectable Melanoma	Unresectable melanoma patients starting anti-PD-1, anti-CTLA4, ± anti-LAG3 mAbs	75 (est)	Prebiotic food-enriched diet (PreFED) PLUS standard of care first-line immunotherapy	Effect of dietary intervention on the abundance of *Faecalibacterium* in stool samples from baseline.
NCT04866810 [87]	The Effect of Diet and Exercise on Immunotherapy and the Microbiome (EDEN)	Melanoma patients starting ipilimumab + nivolumab, relatlimab and nivolumab, pembrolizumab, or nivolumab	24 (act)	High fiber, plant-based diet + exercise prescription with ACT sessions	Feasibility of conducting a decentralized clinical trial involving diet and exercise prescriptions with stool sample collections in patients receiving immunotherapy.

## Data Availability

No new data was created or analyzed in this study. Data sharing is not applicable to this article.

## Abbreviations

The following abbreviations are used in this manuscript:

ANXA1	Annexin A1
BRAF	B-Raf proto-oncogene
cAMP	Cyclic adenosine monophosphate
CCL2	C-C motif chemokine ligand 2 (also known as MCP-1 [Monocyte chemoattractant protein-1])
CREB	cAMP response element-binding protein
CTLA4	Cytotoxic T lymphocyte-associated protein 4
DC	Dendritic cell
EMT	Epithelial-to-mesenchymal
GI	Gastrointestinal
GM	Gut microbiome
GPCR	G protein-coupled receptor
FMT	Fecal microbiota transplant
HAT	Histone acetyltransferase
HDAC	Histone deacetylase
HLA	Human leukocyte antigen
ICB	Immune checkpoint blockade
ICI	Immune checkpoint inhibitor
IFN	Interferon
IL	Interleukin
irAE	Immune-related adverse event
M-CSF	Macrophage colony-stimulating factor
MEK	Mitogen-activated protein kinase kinase (MAP2K) family
MHC	Major histocompatibility complex
mRCC	Metastatic renal cell carcinoma
NF-KB	Nuclear Factor kappa-light-chain-enhancer of activated B cells
NK	Natural killer
NSCLC	Non-small cell lung cancer
OS	Overall survival
PBS	Phosphate-buffered saline
PD-1	Programmed cell death protein 1
PD-L1	Programmed death ligand 1
PFS	Progression-free survival
PRR	Pattern recognition receptor
ORR	Overall response rate
SCFA	Short-chain fatty acid
STING	Stimulator of interferon genes
TILs	Tumor-infiltrating lymphocytes
TMB	Tumor mutational burden
TME	Tumor microenvironment
TNFα	Tumor necrosis factor α
Treg	Regulatory T cell
UK	United Kingdom
UV	Ultraviolet
YGPs	Yeast-derived β-glucan particles
